# The renal manifestations of type 4 familial partial lipodystrophy: a case report and review of literature

**DOI:** 10.1186/s12882-018-0913-6

**Published:** 2018-05-10

**Authors:** Ru-Xuan Chen, Lei Zhang, Wei Ye, Yu-Bing Wen, Nuo Si, Hang Li, Ming-Xi Li, Xue-Mei Li, Ke Zheng

**Affiliations:** 1 Department of Internal Medicine, Peking Union Medical College Hospital, Chinese Academy of Medical Sciences, Beijing, China; 2 Department of Nephrology, Peking Union Medical College Hospital, Chinese Academy of Medical Sciences, Beijing, China; 3 McKusick-Zhang Center for Genetic Medicine, State Key Laboratory of Medical Molecular Biology, Institute of Basic Medical Sciences, Chinese Academy of Medical Sciences and School of Basic Medicine Peking Union Medical College, Beijing, China; 4 Present address: Peking Union Medical College Hospital (East), No. 1 Shuaifuyuan, Dongcheng District, Beijing, 100730 China

**Keywords:** Familial partial lipodystrophy, *PLIN1*, Proteinuria, Focal segmental glomerulosclerosis

## Abstract

**Background:**

Lipodystrophy syndromes are rare disorders of variable body fat loss associated with potentially serious metabolic complications. Familial partial lipodystrophy (FPLD) is mostly inherited as an autosomal dominant disorder. Renal involvement has only been reported in a limited number of cases of FPLD. Herein, we present a rare case of proteinuria associated with type 4 FPLD, which is characterized by a heterozygous mutation in *PLIN1* and has not been reported with renal involvement until now.

**Case presentation:**

A 15-year-old girl presented with insulin resistance, hypertriglyceridaemia, hepatic steatosis and proteinuria. Her glucose and glycated haemoglobin levels were within normal laboratory reference ranges. A novel heterozygous frameshift mutation in *PLIN1* was identified in the patient and her mother. The kidney biopsy showed glomerular enlargement and focal segmental glomerulosclerosis under light microscopy; the electron microscopy results fit with segmental thickening of the glomerular basement membrane. Treatment with an angiotensin-converting enzyme inhibitor (ACEI) decreased 24-h protein excretion.

**Conclusions:**

We report the first case of proteinuria and renal biopsy in a patient with FPLD4. Gene analysis demonstrated a novel heterozygous frameshift mutation in *PLIN1* in this patient and her mother. Treatment with ACEI proved to be beneficial.

## Background

Lipodystrophy syndromes are a group of rare disorders characterized by variable loss of body fat. Adipose tissue loss may affect nearly the entire body (generalized) or only certain regions (partial) or small areas (localized, usually caused by insulin injections). Patients are predisposed to potentially serious metabolic complications associated with insulin resistance, including diabetes mellitus, hypertriglyceridaemia, and steatohepatitis. Other common clinical manifestations include acanthosis nigricans, polycystic ovarian syndrome, hypertension, cardiomyopathy, and arrhythmias. Patients can also have renal dysfunction, which may present as proteinuria, diabetic nephropathy, focal segmental glomerulosclerosis (FSGS) or mesangiocapillary glomerulonephritis (MCGN) [1, 2].

Familial partial lipodystrophy (FPLD) is characterized by loss of subcutaneous fat from the extremities and the trunk. Fat distribution is typically normal in early childhood, followed by onset of partial lipodystrophy around puberty. FPLD is mostly inherited as an autosomal dominant disorder. There are several subtypes of FPLD. Type 2 FPLD (Dunnigan-type, with missense mutations in *LMNA*; OMIM #151660) is the most common, and type 3 FPLD (with heterozygous mutations in *PPARG*; OMIM #604367) is the second most common subtype. Type 2 and type 3 FPLD account for more than 50% of all cases of FPLD [1, 3]. Type 4 FPLD (with heterozygous mutations in *PLIN1*; OMIM #613877) is a rare subtype of FPLD, which is newly recognized and only reported in five unrelated families [4, 5]. This subtype is also designated type 5 FPLD by some scholars [6]. Lipodystrophy is most prominent in the lower limbs and buttocks. The *PLIN1* gene codes for perilipin 1, which is a lipid droplet coat protein predominantly expressed in adipocytes [7]. It is required for optimal lipid incorporation. Here, we present a unique case of renal involvement in an adolescent patient with type 4 FPLD. An unreported heterozygous frameshift mutation in *PLIN1* was identified in the patient and her mother. Until now, no renal involvement has been reported in type 4 FPLD. This is the first case of proteinuria and renal biopsy in a patient with type 4 FPLD.

## Case presentation

A 15-year-old Chinese female was referred to the Department of Nephrology, Peking Union Medical College Hospital because of proteinuria. She was noted to have proteinuria (protein excretion 2–3 g/24 h, maximum 3.1 g/24 h) at the age of 14 years. She had a history of hypertriglyceridaemia (triglyceride, 3.4 mmol/L) and nonalcoholic steatohepatitis (proven by liver biopsy, Grade 1–2, Stage 2 [8]) at the age of 12 years, and insulin-resistant diabetes at the age of 13 years (fasting insulin, 172.4 μU/mL; fasting c-peptide, 9.66 ng/mL; fasting glucose, 5.8 mmol/L; 2-h postprandial glucose, 15.5 mmol/L). Pelvic ultrasound revealed polycystic ovaries, and her serum testosterone concentration was 68 ng/dL. Menarche occurred at the age of 13 years and was regular thereafter. She was told to follow a low-carbohydrate, low-fat diet and was given metformin. With these treatments, her fasting and postprandial glucose level stayed normal, and glycated haemoglobin level was 5.5–5.8%. She had no history of hypertension, and she was a recreational jogger. Her mother, aunt and grandfather had middle-age-onset insulin-resistant diabetes.

**Fig. 1:**
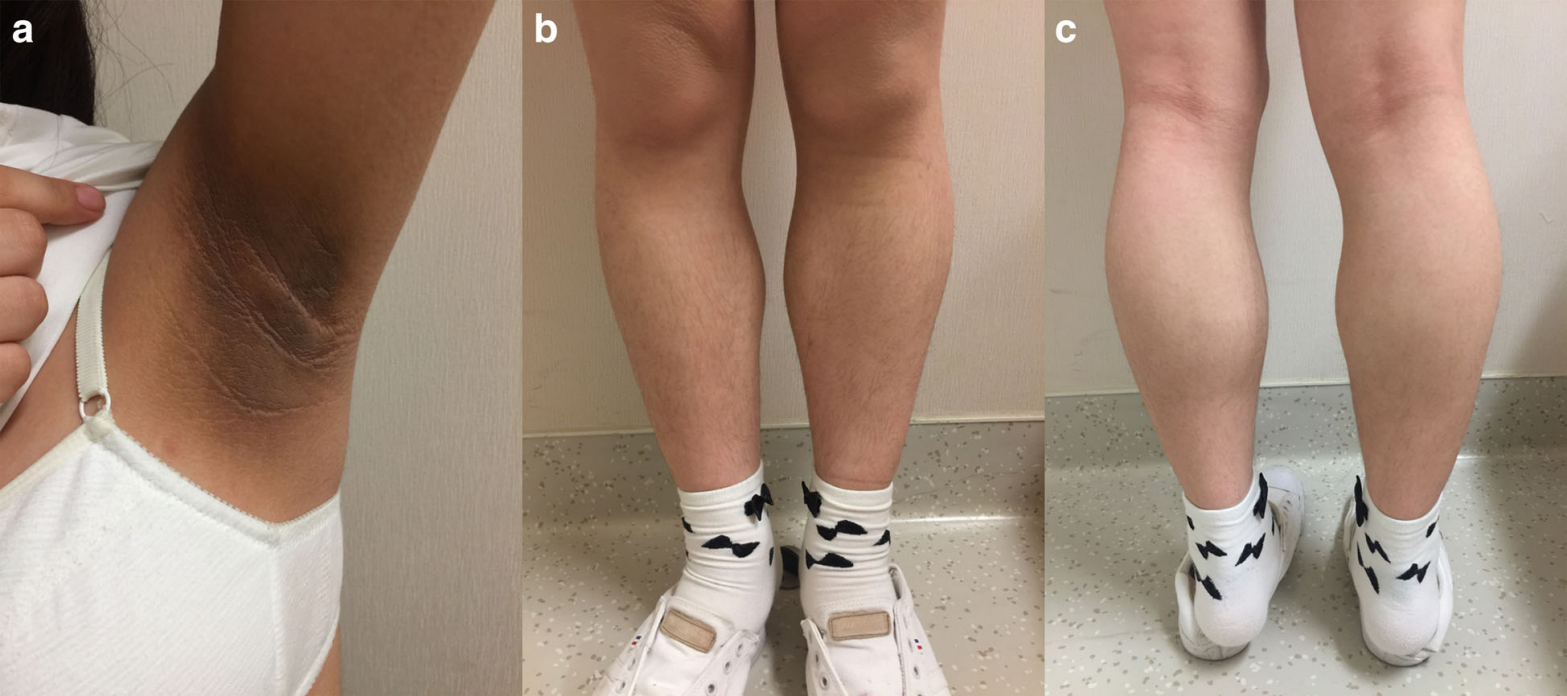
Clinical appearance of the patient. **a** Acanthosis nigricans in the axillary region. **b** Slightly excessive body hair on lower limbs. **c** Muscular appearance of the calf

On admission, her body mass index (BMI, the weight in kilograms divided by the square of the height in metres) was 22.9. Clinical examination revealed prominent muscular appearance of the calves (Fig. 1c). Lipoatrophy was not obvious. Acanthosis nigricans was present in the inguinal, nuchal and axillary regions (Fig. 1a). She had slightly excessive body hair on upper and lower limbs (Fig. 1b).

**Fig. 2:**
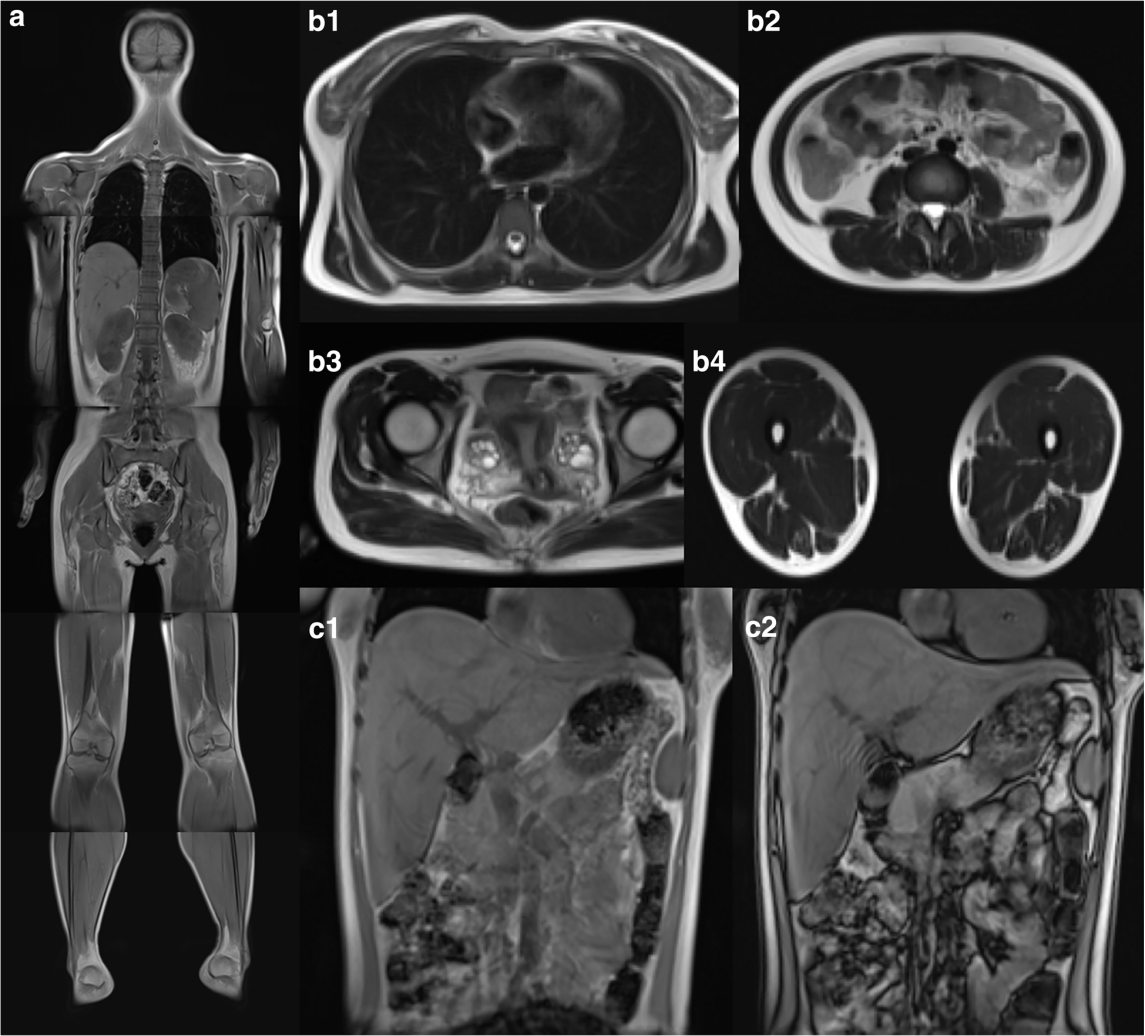
Magnetic resonance images of the patient. Whole body magnetic resonance diffusion-weighted imaging (**a**) demonstrated mildly reduced subcutaneous fat mass. Axial T2-weighted images at the level of breast (**b**1), abdomen (**b**2), pelvic region (**b**3) and thigh (**b**4) illustrated fat distribution of the body and extremities. Image **b**3 revealed polycystic ovaries. Dual phase T1-weighted images (**c**1 and **c**2) showed mild signal loss on out-of-phase images (**c**2), which is consistent with hepatosteatosis

Laboratory evaluation revealed normal serum albumin (47 g/L), decreased creatinine (36 μmol/L), and elevated estimated glomerular filtration rate (154 mL/min/1.73 m^2^, Revised Schwartz Formula). Urine protein excretion was 1.72 g/24 h. Complement components C3 and C4 were 1.630 g/L (range 0.73–1.46 g/L) and 0.166 g/L (range 0.1–0.4 g/L), respectively. Complement factor H was normal, and C3 nephritic factor was not detected. Auto-antibodies and serum immunoglobulins were normal. Renal ultrasound showed both kidneys to be of normal size (length of right kidney was 11.5 cm, length of left kidney was 10.7 cm) and appearance. There was no evidence of diabetic retinopathy or other diabetes-related microvascular complications. Her body fat percentage was 24.7% (TBF-410 Total Body Composition Analyzer, Tanita, Japan). Whole body magnetic resonance diffusion-weighted imaging revealed mildly reduced subcutaneous fat mass, hepatic steatosis, and polycystic ovaries (Fig. 2).

**Fig. 3:**
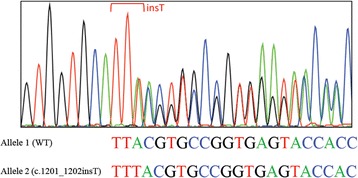
Result of DNA Sanger sequencing analysis of the *PLIN1* gene. Within exon 8, the insertion of thymine in *PLIN1* allele 2 was identified in the patient and her mother but in not her father

Considering the early onset of metabolic abnormalities, whole exome sequencing was performed to detect mutations in the blood samples of the patient and her parents. She and her mother were noted to have a heterozygous insertion of thymine in exon 8 (c.1201_1202insT) of the *PLIN1* gene (Fig. 3), which predicted a frameshift translation leading to the synthesis of 165 aberrant amino acids (p.Y401Lfs*165) and a mutated protein with an aberrant C-terminal. We detected no mutations in genes previously implicated in lipodystrophies (*LMNA, PPARG, CIDEC, LIPE, ADRA2A, AKT2, PCYT1A, AGPAT2, BSCL2, CAV1,* and *PTRF*).

**Fig. 4:**
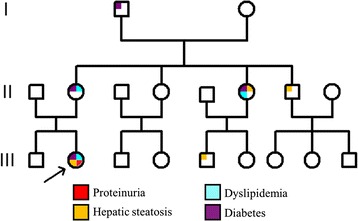
Pedigree of the patient’s family. The patient is designated by the arrow

The BMI of the patient’s mother was 22.1. She did not have acanthosis nigricans. She was noted to have gestational diabetes at the age of 32 years, and she still had diabetes mellitus after the birth of her daughter. While she was on insulin treatment, her fasting glucose was 5.6–7.2 mmol/L. She also had dyslipidaemia (LDL-cholesterol, 4.13 mmol/L; total cholesterol, 5.69 mmol/L; triglyceride, 0.78 mmol/L) treated with diet control and a statin. She had regular health check-ups in recent years. Her urine tests and renal function tests were normal. Ultrasound showed no sign of hepatic steatosis. Her father carried no mutation, and he was generally healthy. He did not have diabetes, dyslipidaemia, fatty liver, or proteinuria. The patient’s maternal grandfather had diabetes. One uncle and one cousin on the maternal side had hepatic steatosis (according to ultrasound results). In addition, one maternal aunt had diabetes, dyslipidaemia and hepatic steatosis (Fig. 4).

**Fig. 5:**
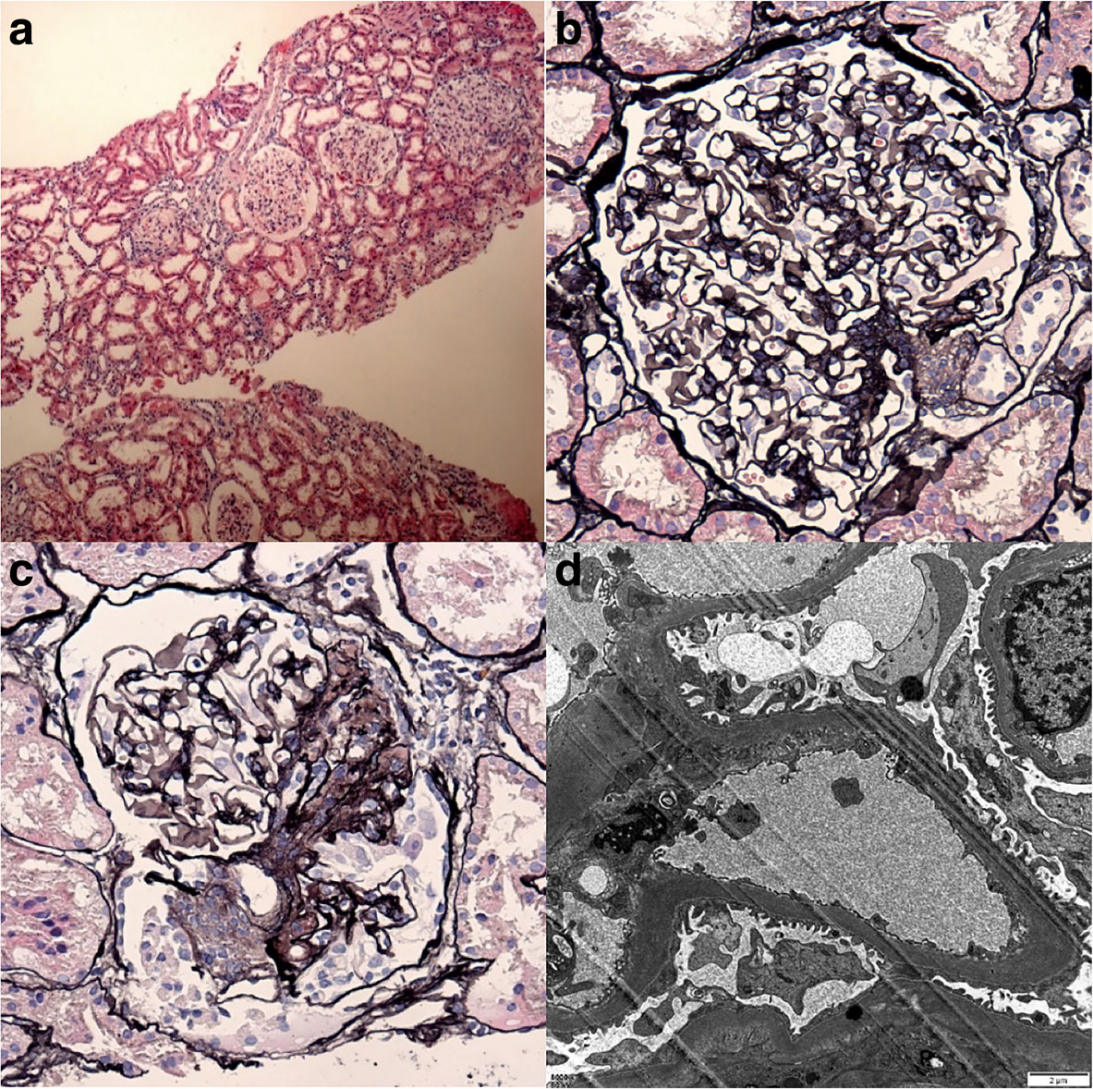
Renal biopsy revealing glomerular lesions under LM and EM. **a** Glomerular enlargement with average glomerular diameter being 230 μm. (LM, H&E stain, × 40). **b** Mild mesangial hypercellularity, without significant matrix expansion. (LM, PASM, stain, × 400). **c** Focal sclerosis of 1 glomerulus, and exudative lesions were concomitant. (LM, PASM stain, × 400). **d** Segmental thickening of glomerular basement membrane and mild effacement of the podocyte foot processes, mild mesangial matrix expansion. (EM, × 8000)

Renal biopsy was performed on the patient. Immunofluorescence (IF) microscopy showed no immunoglobulin deposits. Light microscopy (LM) revealed that in the absence of significant background tubulointerstitial damage, 1 out of 17 glomeruli obtained was globally sclerosed, 2 glomeruli displayed segmental sclerosis (one segmental sclerosis adhered to Bowman’s capsule and involved the perihilar region, the other one was concomitant with exudative lesion (Fig. 5c)), and the remaining glomeruli displayed mild mesangial hypercellularity without matrix expansion (Fig. 5b). Glomerular enlargement was observed (Fig. 5a), with average glomerular diameter being 230 μm and maximum diameter being 251 μm. Transmission electron microscopy (EM) revealed focal thickening of the glomerular basement membrane (GBM) and mild focal effacement of foot processes; mild expansion in mesangial matrix was also shown under EM (Fig. 5d); no electron-dense deposits were present.

She was given benazepril (10 mg per day, gradually titrated to 20 mg twice a day) and metformin. Benazepril was well-tolerated, with no cough, hypotension or hyperkalaemia. After 1 month, her renal protein excretion was decreased to 0.81 g/24 h. Serum creatinine level was 40 μmol/L.

## Discussion

The estimated prevalence of FPLD is 1 in 1,000,000 people in the general population. FPLD is more common in Caucasians (79%) and has a female predominance (83%) [6].

Type 4 FPLD is a rare autosomal dominant disorder caused by mutations in the *PLIN1* gene leading to defective adipogenesis and dysregulated lipolysis. To date, only five families with type 4 FPLD have been reported, and no case of type 4 FPLD has been reported in Asia.

The *PLIN1* gene codes for perilipin 1, which is the most abundant phosphoprotein in adipocytes. Perilipin 1 associates with the phospholipid surface layer of the lipid droplet and is critical for optimal lipid metabolism [7]. It regulates lipid accumulation and release from the lipid droplets primarily through the regulation of lipases, including adipose tissue triglyceride lipase (ATGL) and hormone-sensitive lipase (HSL). ATGL and HSL catalyse the hydrolysis of tri- and diacylglycerol, releasing fatty acids and monoacylglycerol [9]. Perilipin-null mice were lean and resistant to obesity and exhibited increased basal lipolysis but diminished catecholamine-stimulated lipolysis [10]. Loss-of-function mutations in the *PLIN1* gene were reported in patients with type 4 FPLD [4, 5]. Type 4 FPLD is an autosomal dominant disorder. All reported mutations were heterozygous frameshift mutations affecting the COOH-terminus of perilipin 1, leading to the failure of the proteins to prevent ABHD5 (αβ-hydrolase domain containing 5) from activating ATGL [4, 11]. One mutant (439 fs mutant) had a lower expression level and shorter half-life than the wild-type protein [4], and the other two (404 fs and 398 fs mutants) failed to bind ABHD5 [11]. Patients with *PLIN1* mutations reportedly had higher basal lipolytic rates and smaller adipocytes with increased infiltration of macrophages and fibrosis in the adipose tissue [4, 5]. Increased plasma fatty acids can lead to lipid accumulation in ectopic sites, such as the liver and skeletal muscle, where they are instrumental in causing insulin resistance [12].

In this case, we identified a novel heterozygous frameshift mutation (c.1201_1202insT) affecting the carboxy-terminus (401 fs) of perilipin 1. The mutation site was between two previously reported mutations (c.1191_1192delAG, and c.1308-1309delTG) [4, 5]. Amino acids 380–427 in the human sequence of perilipin 1 were shown to be crucial for ABHD5 binding [13]. We thus inferred that this p.Y401Lfs*165 mutant could not effectively bind ABHD5 and was likely to be pathogenic. The fact that the patient’s mother (a carrier of this mutation) was affected, while the patient’s father (without this mutation) was generally healthy, was supportive of the pathogenicity of this mutation. Furthermore, this heterozygous frameshift variant had not been reported in databases of the general population including GNOMAD (genome aggregation database), ExAC (exome aggregation consortium), 1000 Genomes, and dbSNP (single nucleotide polymorphism database).

As adipose tissues have pivotal roles in maintaining healthy metabolic homeostasis, FPLD patients usually have metabolic complications including insulin resistance, diabetes, dyslipidaemia and hepatic steatosis [1]. The severity of the metabolic complications may be roughly proportional to the extent of lipoatrophy. However, in this case, the patient had prominent insulin resistance, dyslipidaemia and hepatic steatosis, but only mildly reduced subcutaneous fat. The patient’s mother, who carried the same heterozygous frameshift mutation, had milder symptoms. She only had diabetes and mild dyslipidaemia. Unfortunately, we did not have the opportunity to assess the mother’s fat mass and fat distribution. However, her body type appeared normal. Possible explanations for the variability of the phenotype of this mutation in this family include interindividual variability in the gene expression.

Renal dysfunction of lipodystrophies (familial and acquired) can present as proteinuria, diabetic nephropathy, FSGS or MCGN [1, 2]. Different renal pathologies have been recognized in cases of generalized lipodystrophy (familial or acquired) [14], and renal biopsy revealed FSGS in four patients, MCGN in two patients, and diabetic nephropathy in one patient. The majority treated with recombinant leptin demonstrated a reduction in proteinuria and hyperfiltration. Approximately 22% of patients with acquired partial lipodystrophy developed MCGN type II (dense deposit disease) after a median of approximately 8 years following the onset of lipodystrophy [15]. These patients usually had low plasma C3 and elevated C3 nephritic factor; the latter was thought to cause lysis of adipose tissue [16]. The first case of biopsy-proven MCGN type II associated with FPLD was reported in a case of type 2 FPLD (with mutation in the *LMNA* gene), with no hypocomplementaemia or evidence of C3 nephritic factor [17]. The mechanism underlying the association was unknown. Proteinuria and end-stage renal failure had been reported in type 2 FPLD before [18], but without renal biopsy, they were assumed to be secondary to diabetic nephropathy.

Although the majority of FSGS cases are idiopathic, a number of genetic and familial forms of FSGS have been recognized, with mutations in genes encoding proteins integral to basement membrane and/or glomerular podocyte function. Thong KM et al. reported a large pedigree of type 2 FPLD (with mutation in the *LMNA* gene), in which four members were found to have biopsy-proven FSGS [19]. No immune complexes were observed, and there were no features suggesting diabetic nephropathy on biopsy. The maximum glomerular diameters of four patients were 142 μm, 200 μm, 206 μm and 218 μm. The *LMNA* gene codes for lamins A/C, major components of the nuclear lamina which function in nuclear architecture, integrity and the regulation of gene expression [20]. Since there was no evidence of secondary causes of FSGS, the association between FSGS and type 2 FPLD suggested the physiological role of *LMNA* could be relevant to the maintenance of glomerular structure and function [19].

Until now, no case of renal involvement has been reported in type 4 FPLD. This is the first case of proteinuria and renal biopsy in a patient with type 4 FPLD. In this case, the average glomerular diameter was 230 μm, suggestive of glomerulomegaly. According to a study by Samuel T et al., in histologically normal kidneys of 24 males (20–69 years), the mean (SD) glomerular diameter was 201 (28) mm (range 110–276 mm) [21]. Glomerulomegaly could be the result of glomerular hyperfiltration, which was caused by the abnormal metabolic status in the patient [22]. Our patient’s estimated glomerular filtration rate was 154 mL/min/1.73 m^2^. Adaptive FSGS may be associated with excessive nephron workload in hyperfiltration status. With glomerular hypertrophy, pressure and flow in the glomeruli are increased, leading to podocyte stress (a mismatch between glomerular load and glomerular capacity). Podocytes compensate by hypertrophy to cover more of the glomerular capillary surface. Increased mechanical distension and shear force on podocytes can be a critical factor driving podocyte injury [23]. In the EM results of this patient’s renal biopsy tissue, mild foot process fusion and focal GBM thickness were evidence of the above hypothetical pathogenesis. In addition, the serum albumin level of the patient is normal, which is common in adaptive FSGS while unusual in primary FSGS. Adaptive FSGS usually responds well to RAAS (renin–angiotensin–aldosterone system) antagonism, often with > 50% proteinuria reduction [24]. RAAS antagonism directly addresses the haemodynamic alterations in adaptive FSGS. Combining this patient’s clinical features, pathological findings and response to ACEI, we deduced that the pathogenesis of this patient’s renal lesion was similar to obesity-associated FSGS, which was a maladaptive response to the renal haemodynamic changes [25]. However, given the patient’s history of insulin-resistant diabetes, the early stages of diabetic nephropathy (Class I, Renal Pathology Society classification, [26]) should be carefully watched for.

Regarding the patient, after 1 month of treatment with benazepril, her renal protein excretion was decreased to 0.81 g/24 h. Serum creatinine level was 40 μmol/L. Estimated glomerular filtration rate was 139 mL/min/1.73 m^2^ (Revised Schwartz Formula). Apart from RAAS antagonism, management of metabolic abnormalities is also very important. Diet and exercise form an integral part of the treatment plan. Metformin and long-chain n-3 polyunsaturated fatty acids help alleviate insulin resistance and dyslipidaemia.

There are some limitations of our approach to this case. First, we only performed gene sequencing of the patient and her parents, not the entire family, because her brother was abroad and other relatives were also remote. The mutation detected in the patient and her mother was novel. We only predicted the function of the mutated gene based on existing knowledge. The co-segregation analysis of the pathogenesis of this mutation was limited. Second, the follow-up period was only 1 month. We will continue to follow the case in the future.

## Conclusion

FPLD is a group of rare disorders. To date, no case of type 4 FPLD has been reported to have kidney disease. We reported here the first case of a type 4 FPLD patient presenting with proteinuria. The renal biopsy revealed features of glomerulomegaly and adaptive FSGS secondary to glomerular hyperfiltration. Treatment with an ACEI helped decrease renal protein excretion. For young patients who present with early-onset proteinuria, FPLD should be considered in the differential diagnosis.

## Abbreviations

ABHD5: αβ-hydrolase domain containing 5
ACEI: Angiotensin-converting enzyme inhibitor
ATGL: Adipose tissue triglyceride lipase
BMI: Body Mass Index
EM: Electron microscopy
FPLD: Familial partial lipodystrophy
FSGS: Focal segmental glomerulosclerosis
H&E: Haematoxylin and eosin stain
HSL: Hormone-sensitive lipase
IF: Immunofluorescence
LM: Light microscopy
MCGN: Mesangiocapillary glomerulonephritis
PASM: Periodic Schiff-methenamine stain
RAAS: Renin–angiotensin–aldosterone system
